# Evaluation of the Use of Formalin-Fixed and Paraffin-Embedded Archive Gastric Tissues for Microbiota Characterization Using Next-Generation Sequencing

**DOI:** 10.3390/ijms21031096

**Published:** 2020-02-07

**Authors:** Ines Pinto-Ribeiro, Rui M. Ferreira, Joana Pereira-Marques, Vanessa Pinto, Guilherme Macedo, Fátima Carneiro, Ceu Figueiredo

**Affiliations:** 1 Institute of Molecular Pathology and Immunology of the University of Porto (Ipatimup), 4200-135 Porto, Portugal; 2 Instituto de Investigação e Inovação em Saúde, Universidade do Porto (i3S), 4200-135 Porto, Portugal; 3 Faculty of Medicine of the University of Porto (FMUP), 4200-319 Porto, Portugal; 4 Department of Gastroenterology, Centro Hospitalar Universitário S. João, 4200-319 Porto, Portugal; 5 Department of Pathology, Centro Hospitalar Universitário S. João, 4200-319 Porto, Portugal

**Keywords:** microbiota, formalin-fixed and paraffin-embedded tissues, 16S rRNA gene, next-generation sequencing

## Abstract

Large numbers of well-characterized clinical samples are fundamental to establish relevant associations between the microbiota and disease. Formalin-fixed and paraffin-embedded (FFPE) tissues are routinely used and are widely available clinical materials. Since current approaches to study the microbiota are based on next-generation sequencing (NGS) targeting the bacterial 16S rRNA gene, our aim was to evaluate the feasibility of FFPE gastric tissues for NGS-based microbiota characterization. Analysis of sequencing data revealed the presence of bacteria in the paraffin control. After the subtraction of the operational taxonomic units (OTUs) present in the paraffin control to the FFPE tissue sample dataset, we evaluated the microbiota profiles between paired FFPE and frozen gastric tissues, and between different times of archiving. Compared with frozen gastric tissues, we detected a lower number of OTUs in the microbiota of paired FFPE tissues, regardless of the time of archiving. No major differences in microbial diversity were identified, but taxonomic variation in the relative abundance of phyla and orders was observed between the two preservation methods. This variation was also evident in each case and throughout the times of FFPE archiving. The use of FFPE tissues for NGS-based microbiota characterization should be considered carefully, as biases can be introduced by the embedding process and the time of tissue archiving.

## 1. Introduction

The collection of microorganisms (bacteria, archaea, viruses, and fungi) that colonize humans in each anatomic site is known as the microbiota. A microbiome includes microorganisms (microbiota), their genomes, and their surrounding environment [1]. The microbiome plays an important role in the maintenance of human health, and initiatives such as the Human Microbiome Project have characterized the bacterial microbiota of healthy individuals across six different sites in the human body [2]. Currently, there is evidence that alterations to the normal composition of the microbiome are involved in the development of a wide range of diseases, including obesity, inflammatory bowel diseases, and cancer [3,4,5]. However, the lack of agreement between publications concerning the major groups of bacteria responsible for the disease phenotypes highlights the growing need for large studies in order to validate the results [5,6,7,8].

The reliable description of the microbiome depends on the availability of a large number of well-characterized and well-preserved clinical specimens. Frozen tissue samples are the preferable type of specimens to study the microbiota, since they make it possible to isolate high-quality nucleic acids and to undertake consistent molecular testing [9]. Formalin-fixed, paraffin-embedded (FFPE) tissues are widely available clinical materials in pathology departments’ archives, as they are routinely prepared for histopathological analyses [10]. FFPE samples are easy to handle, suitable for long-term storage, and can be used in a wide range of molecular applications, such as quantitative polymerase chain reactions (qPCRs), whole-genome sequencing, and transcriptomics [5,11,12,13]. Therefore, FFPE specimens could be thought of as an alternative for microbiota profiling in large-scale studies.

The microbiota can be characterized by various technical approaches. Next-generation sequencing (NGS) targeting the bacterial 16S rRNA gene is one of the most commonly-used techniques [14,15]. The feasibility of the microbiota characterization using NGS of the 16S rRNA gene in FFPE specimens has not been investigated in detail. In fact, only a few studies haveused FFPE tissue specimens to explore the microbiota composition in mucosal samples [16,17,18,19]. However, they lack important aspects such as paired comparisons with fresh tissues and controls for contamination and time of archiving. In this scenario, our aim was to evaluate the use of FFPE tissues for NGS-based microbiota profiling. We evaluated the effect of the embedding and the time of archiving of the specimens by comparing the microbiota composition of paired frozen and FFPE tissues after 6, 12, and 18 months of archiving.

## 2. Results

### 2.1. Impact of the Embedding Process of FFPE Samples on the Microbiota Profiles

We studied 20 samples obtained from five different cases. From each case, two adjacent fragments of tissue were used: one was frozen, and the other was paraffin embedded. From each paraffin block, a sample was obtained at 6, 12, and 18 months post-archiving. All samples were characterized for their microbiota composition by 16S rRNA gene sequencing.

**Figure 1:**
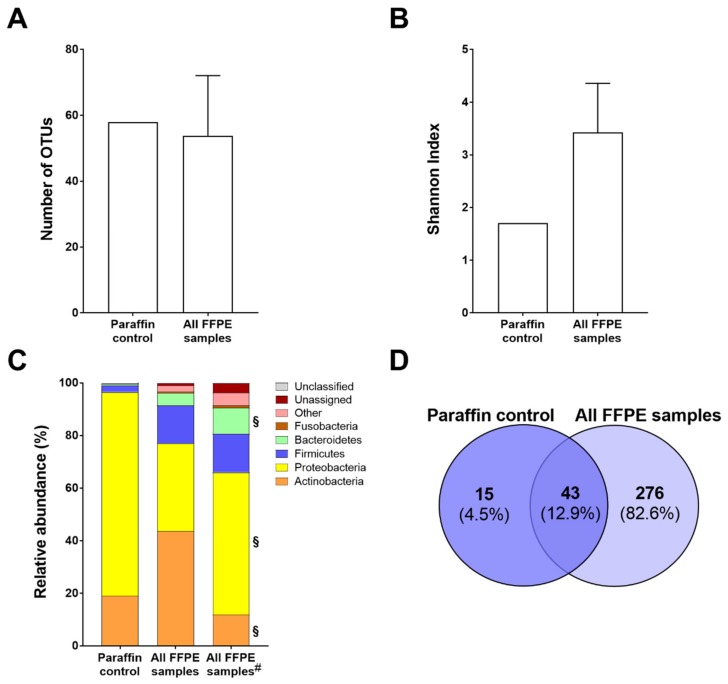
Impact of the embedding of FFPE tissues on the microbiota characterization. (**A**) Number of OTUs of the paraffin control and all FFPE samples obtained after randomly subsampling to a fixed depth. (**B**) Microbial alpha-diversity assessed by the Shannon index. (**C**) Taxonomic profile at the phylum level discriminating the most abundant phyla in the paraffin control and all FFPE samples before and after (#) OTU subtraction. § stands for significantly different from all FFPE samples at *p* < 0.05. (**D**) Venn diagram showing the number OTUs common and exclusive to the paraffin control and FFPE samples.

Since FFPE tissues are prepared routinely in nonsterile conditions, we started by evaluating the influence of the embedding process of tissue samples on microbiota profiling. After sequencing and quality filtering, a mean of 62,920 reads and 56 operational taxonomic units (OTUs) were identified per sample. Variation of the sequencing depth was controlled by randomly subsampling reads to a fixed depth, resulting in a similar mean number of OTUs (54 OTUs; *p* = 0.7434; Figure 1A). In the paraffin control, which consisted of a similarly processed paraffin block without tissue, 62,095 reads and 58 OTUs were initially identified (Figure 1A and Supplementary Figure S1). The number of OTUs per sample is shown in Supplementary Figure S1. The presence of bacteria in the paraffin control sample was confirmed by the use of a universal qPCR assay directed to the 16S rRNA gene (Supplementary Figure S1). 

The microbial community present in this control sample had a lower Shannon index of diversity than that globally present in the FFPE tissues (Figure 1B). Individually, the majority of FFPE tissue samples had higher diversity than the control sample (Supplementary Figure S1). The majority of FFPE tissue samples also had microbial communities that were considerably distant from those present in the paraffin control sample, as measured by the weighted Unifrac metrics (Supplementary Figure S1).

Since bacteria were detected in the paraffin control sample, we characterized all samples at the taxonomic level. The bacteria detected in the paraffin control were mostly of the phyla Proteobacteria (77.4%) and Actinobacteria (19.1%), whereas the microbiota globally present in the FFPE tissues had a different order of mean relative abundance (Actinobacteria: 43.6%, Proteobacteria: 33.4%, and Firmicutes: 14.5%; Figure 1C and Supplementary Table S1). The results of the relative abundance of phyla per sample are shown in Supplementary Figure S1.

Aiming to identify the taxa that were present in the paraffin control and that could be masking the microbiota profile of the FFPE tissue samples, we performed an OTU-based analysis. We identified 15 OTUs exclusive to the paraffin control and 43 OTUs (12.9% of all OTUs) shared between the tissues and the paraffin control (Figure 1D). Of note, four of the shared OTUs (OTU2, OTU1, OTU20, and OTU4) represented 93% of the total bacteria abundance found in the paraffin control (Supplementary Figure S1). Therefore, for the subsequent analyses, the 58 OTUs aforementioned were subtracted from the microbial datasets of all FFPE tissue samples. After OTU subtraction, the relative abundance of Proteobacteria (54.1%) and Bacteroidetes (9.9%) significantly increased (*p* = 0.001 and *p* = 0.021, respectively), while the abundance of Actinobacteria decreased (11.9%; *p* < 0.0001; Figure 1C and Supplementary Table S1).

These results demonstrate that the paraffin embedding process introduces a bias that influences the profile of the microbiota present in FFPE tissue samples.

### 2.2. Comparison between Paired FFPE and Frozen Tissues and the Impact of Time of Archiving on the Microbiota Profiles

We next evaluated whether the structure and the composition of the microbiota of FFPE tissues resembled those of the respective paired frozen tissues, and whether the time of archiving influenced the characterization of the microbiota. For these analyses, we considered only the microbiota of FFPE tissue samples after OTU subtraction.

**Figure 2:**
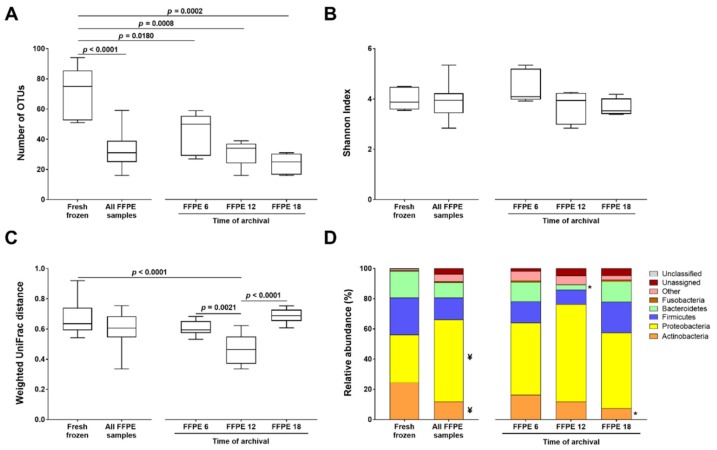
Comparison of the microbiota profiles between frozen and FFPE tissues and between different times of FFPE tissue archiving (**A**) Number of OTUs in each group of samples. (**B**) Microbial alpha-diversity assessed by the Shannon index. (**C**) Microbial beta-diversity calculated by the weighted UniFrac distances. (**D**) Relative abundance of phyla in frozen tissues, all FFPE tissue samples, and FFPE tissues archived for 6, 12, and 18 months. ¥ and * stand for significantly different from frozen tissues at *p* < 0.05.

A significantly higher median number of OTUs was observed in the microbiota of frozen tissues (n = 75) than in all FFPE tissues (n = 31; *p* < 0.0001) and in the FFPE tissues archived for 6 (n = 50; *p* = 0.011), 12 (n = 34; *p* = 0.0008), and 18 (n = 25; *p* = 0.0002) months (Figure 2A).

No significant differences were detected regarding the microbial alpha-diversity between the frozen and FFPE tissues globally, or those obtained at 6, 12, or 18 months post-archiving (Figure 2B). Overall, no significant differences were observed in the microbial beta-diversity between the two preservation methods (Figure 2C). The microbial community present in FFPE tissues obtained 12 months post-archiving was separated from the communities of frozen (*p* < 0.001) and also FFPE tissues preserved for 6 and 18 months (*p* = 0.002 and *p* < 0.001, respectively; Figure 2C). The microbiota of FFPE tissues obtained 6 and 18 months post-archiving, however, could not be distinguished from that of the frozen tissues.

Taxonomically, and taking all FFPE samples together, there were differences at the phylum level between the microbiota of the tissues preserved by the two methods (Figure 2D and Supplementary Table S1). Compared with the frozen tissues, the microbiota of the FFPE tissues had a statistically significant increase in the mean relative abundance of Proteobacteria (*p* = 0.042) and a decrease in Actinobacteria (*p* = 0.033). No significant differences were observed in the relative abundances of Firmicutes, Bacteroidetes, and Fusobacteria.

By comparing frozen with their paired FFPE tissues obtained after 6, 12, and 18 months of archiving, we observed statistically significant decreases of Bacteroidetes at 12 months (*p* = 0.039) and Actinobacteria at 18 months (*p* = 0.045) postarchiving (Figure 2D and Supplementary Figure S2). These differences were more evident at the order level (Supplementary Figure S2). 

When examining the taxonomic composition per case, we observed a relatively consistent profile at the phylum level according to the time of archiving, while at the order level, the differences became more noticeable (Supplementary Figure S2).

Taken together, our results provide evidence that the profile of the microbiota of tissues included in paraffin does not completely resemble the profile of their paired frozen samples. Furthermore, the time of archiving had an effect on the microbiota composition.

## 3. Discussion

We evaluated the feasibility of using FFPE tissue specimens for characterizing the microbiota by NGS of the 16S rRNA gene, using gastric tissue specimens as a model. First, we demonstrated that it is essential to include a control of the embedding process, consisting of a block of paraffin without tissue, in order to distinguish the bacteria associated with the paraffin inclusion from the microbial signatures of the tissue. The embedding provides support for the tissue, which is essential for thin section cutting of tissue to be used in the histopathological analysis. In agreement with the fact that this process was performed under nonsterile conditions, our results showed that the paraffin control contained bacteria. The bacterial community in the paraffin control had low diversity, was distinct from that present in FFPE tissues, and was constituted mainly of bacteria of the Proteobacteria and Actinobacteria phyla. These findings are highly suggestive that the paraffin embedding process introduces a bias in the profile of the microbiota of FFPE tissue specimens.

As currently there are no specific guidelines to overcome this type of bias in NGS data analysis, we subtracted the OTUs of the paraffin control to the full FFPE dataset. This subtraction led to significant taxonomic alterations in the order of relative abundance of taxa in the FFPE tissues. In fact, only a few studies have used FFPE tissues to characterize the microbiota by NGS, and none of them controlled for the embedding process [16,17,18,19].

The feasibility of using FFPE tissues was further analyzed by comparing the microbiota profiles of the FFPE and their paired frozen tissues, and by evaluating the influence of the time of archiving on the microbial profiling. A striking decrease was observed in the number of OTUs that could be detected in the microbiota of FFPE tissues, regardless of the time of archiving. Overall, there were no differences in alpha- or beta-diversity between the two methods of sample preservation. Yet, the microbial community present in FFPE tissues characterized 12 months postarchiving was separated from the communities of frozen and FFPE tissues preserved for 6 and 18 months. Taxonomically, there was variation in the relative abundance of taxa between frozen and FFPE tissues, which could be detected already at the phylum level, but was more striking at lower taxonomic levels. Both FFPE and frozen tissues underwent similar processing regarding DNA isolation, PCR, and sequencing. Therefore, differences in the microbial composition between the two preservation methods might be attributed to the deamination of cytosine residues, which may result in single-nucleotide variants, and to DNA degradation that occurs during formalin fixation [20,21,22]. In addition, the low amount of tissue present in the paraffin cuts may also limit our ability to detect the bacteria in the tissue.

Supporting our findings, a study that compared the microbiota composition between paired frozen and FFPE brain specimens in a small cohort of Alzheimer’s cases also reported differences in the taxonomic profile between the two types of preservation method [18]. Another recent study used FFPE tissues to investigate bacterial involvement in colorectal carcinogenesis by qPCR and, in a subset of cases, by 16S rRNA gene NGS [17]. Although the authors did not compare their NGS results with those of the paired frozen tissues, they reported agreement between their findings and previously published data. Nevertheless, they also show that their NGS data did not clearly correlate with qPCR results. 

In our study, increased time of archiving of the tissues also influenced the taxonomic characterization of the microbiota, as FFPE tissues preserved for 12 and 18 months had progressively higher differences. The consistency of the microbiota analysis over time is highly relevant, since this type of clinical material can be stored for decades at low cost, allowing the study of large numbers of samples. In keeping with our data, in spite of the differences in context, a study that examined the potential use of FFPE tissues from different human organs for exome and transcriptomics NGS analyses also identified dissimilarities associated with the time of archiving of FFPE specimens [12]. Specifically, the authors showed that FFPE specimens which had been stored for less than 3 years shared 70%–80% of genetic variants with their paired frozen tissue, while those archived for more than 3 years had a higher percentage of exclusive genetic variants. Nevertheless, a study that evaluated the robustness of whole-exome sequencing on FFPE tissues of high-grade ovarian serous adenocarcinoma stored 3–32 years demonstrated that those samples could still provide usable data, regardless of storage time [11].

Although our study contains a limited number of samples and controls, to the best of our knowledge, it is the first to evaluate the feasibility of FFPE specimens for microbiota profiling that includes paired frozen and FFPE tissues and a control of the embedding process. Future studies should consider the inclusion of additional negative controls to ascertain possible biases introduced during the embedding process, and the treatment with uracil N-glycosylase prior to sequencing, to reduce possible deamination of cytosine residues. Additionally, comparisons between the microbiota profiles obtained by the use of different hypervariable regions of the 16S rRNA gene may be useful for further method standardizations.

In conclusion, the use of FFPE tissues to profile the tissue-associated microbiota should be considered with extreme caution, as the bacteria community detected in FFPE tissues does not fully recapitulate that of frozen tissues.

## 4. Materials and Methods

### 4.1. Samples

Fragments of non-neoplastic gastric tissues were collected from five cases at the Tissue and Tumor Bank of the Centro Hospitalar Universitário S. João, Porto, Portugal. From each case, two fragments were collected: one was immediately snap frozen in liquid nitrogen, and the other was fixed in 10% neutral buffered formalin and embedded in paraffin. The study was approved by the institutional review board (no. 76-16, 07-04-2016). Samples were delinked and unidentified from their donors.

### 4.2. DNA Extraction

Initially, each block was trimmed to expose the tissue surface obtaining a representative section to be cut. Afterward, from each paraffin block, four cuts of 10 μm of FFPE specimens were obtained at 6, 12, and 18 months postarchiving. To avoid cross-contamination, the blade was changed after each block. The control paraffin block without tissue was processed similarly.

DNA was isolated from FFPE and their paired frozen tissues using the QIAamp DNA FFPE Tissue Kit (Qiagen, Hilden, Germany) following the manufacturer’s instructions. Initially, paraffin was removed by xylene treatment followed by centrifugation from FFPE samples, while frozen samples were homogenized with PBS 1x and Buffer ATL using the TissueRuptor (Qiagen). Then, all tissues were lysed under denaturing conditions with proteinase K at 56 °C. To avoid RNA contamination, samples were treated with RNase A (Grisp, Porto, Portugal). Afterwards, DNA was bound to a QIAamp MinElute column and washed to eliminate contaminants. Finally, preheated Microbial DNA-free water (Qiagen) was used to elute DNA from the membrane of the columns.

### 4.3. Real-Time Quantitative Polymerase Chain Reaction (qPCR)

The total bacterial load present in each sample was evaluated using a universal 16S rRNA gene qPCR assay that comprised primers targeting a conserved region flanking the V5-V6 regions. qPCR reactions were prepared containing 1× NZY qPCR Green Master Mix, ROX (NZYtech, Lisbon, Portugal), 1 μM of forward (U789F_v56, 5′-TAGATACCCBDGTAGTCC-3′) and reverse (U1053R_v56, 5′-CTGACGACRRCCATGC-3′) primers (Integrated DNA Technologies, Heverlee, Belgium), Microbial DNA-free water (Qiagen), and 1 μL of DNA. The qPCR was performed in a 7500 Fast Real-Time PCR System (Applied Biosystems, Foster City, CA, USA) with the following conditions: 2 min at 50 °C, 10 min at 95 °C, followed by 35 cycles of denaturation at 95 °C for 15 s and annealing/extension at 62 °C for 1 min. The amplification steps were followed by a melt dissociation step to check for nonspecific product formation. This step comprised an additional cycle of 95 °C for 15 s, 60 °C for 1 min, 95 °C for 30 s, and 60 °C for 15 s. In addition, the PCR product purity was also controlled by 2% agarose gel electrophoresis. Two replicates were performed for each sample. To exclude any potential environmental contaminant in qPCR reactions, blanks were prepared using Microbial DNA-free water (Qiagen) instead of DNA. The relative standard curve method was used to quantify the total bacterial load of paired frozen and FFPE specimens. To create standard curves, the full 16S rRNA gene of *Escherichia coli* was cloned into the pGEM-T easy vector system (Promega, Madison, WI, USA). Dilution series of known plasmid copy numbers was used to create a standard curve by plotting the log10 of copy numbers of 16S rRNA gene in the dilution series against the determined threshold cycle value. To control the calibration of standard curve, the reaction parameters were obtained including slope, y-intercept, correlation coefficient, and efficiency.

### 4.4. 16S rRNA Gene Amplification and Sequencing

The V5-V6 hypervariable regions of the 16S rRNA gene were amplified using universal primers fused with Illumina adapters sequences U789F_v56_ngs 5′-CGTCGGCAGCGTCAGATGTGTATAAGAGACAGTAGATACCCBDGTAGTCC-3′ and U1053R_v56_ngs 5′-GTCTCGTGGGCTCGGAGATGTGTATAAGAGACAGCTGACGACRRCCATGC-3′ (Integrated DNA Technologies). The PCR reactions were performed in 35 μL 1x AmpliTaq Gold 360 Master Mix (Applied Biosystems, Foster City, CA, USA) and 0.4 μM of forward and reverse primers (Invitrogen, Foster City, CA, USA). Microbial DNA-free water (Qiagen) was added to PCR negative controls instead of DNA. Amplicons underwent a purification step with magnetic beads using the Axy Prep PCR Clean-Up Kit (Axygen, Union City, CA, USA) and visualized in 1.5% agarose gels. Their concentration was determined with the Qubit dsDNA HS Assay Kit (Life Technologies, Foster City, CA, USA). Equal amounts of amplicons were used for sequencing library construction using the Illumina 16S Metagenomic Sequencing Library preparation protocol. The final sequencing library was sequenced with MiSeq Reagent Kit v3 (Illumina, San Diego, CA, USA) in the Illumina MiSeq platform, using 300 bp paired-end sequencing reads with an expected output of 100,000 reads per samples.

### 4.5. Sequencing Data Analysis

Raw paired-end reads were assembled and primers sequences were removed at both ends. Reads were quality filtered by imposing a maximum number of expected errors of 1.0 and trimmed to a fixed length (29,000 reads). Filtered reads were dereplicated and clustered into OTUs using the UPARSE algorithm [23], which builds OTUs and performs chimera filtering. As quality control, the constructed OTUs were aligned against two databases, the 18S rRNA (SILVA 18S rRNA v123) and the internal transcribed spacer (RDP ITS v2) sequences in order to search for eukaryotic contaminations. An OTU table was constructed by mapping reads back to OTUs to get counts per sample. Furthermore, each OTU was taxonomically assigned using the SINTAX algorithm [24] with 16S RDP Classifier training set v16 as the reference database. OTUs were classified as follows: “Other” includes the phyla or orders not mentioned in the graph; “unassigned” comprises OTUs not classified at the phylum or order level; and “unclassified” identifies nonbacterial species. Alpha-diversity was determined by the Shannon index. Beta-diversity was assessed by the weighted UniFrac distance [25]. Differences in beta-diversity were calculated in distance matrices and visualized in boxplots. Sequencing data analysis was performed using usearch_v8.1.11861_i86linux64 and QIIME v1.9 [26].

### 4.6. Statistical Analysis

The GraphPad Prism software (v. 7.04, GraphPad Software Inc., La Jolla, CA, USA) was used to perform the statistical analysis. The D’Agostino Pearson omnibus and Shapiro-Wilk normality tests were used to test whether the data followed a Gaussian distribution. Differences in the number of OTUs, alpha and beta diversity between frozen tissue, and all FFPE samples were evaluated using Student’s t-test, while one-way analysis of variance followed by Holm-Sidak’s multiple comparisons test was used to compare frozen tissue with FFPE samples obtained at 6, 12, and 18 months. The relative abundances of the most abundant taxa were compared between frozen tissue and all FFPE samples by the Mann-Whitney test, and the Kruskal-Wallis nonparametric test corrected with Dunn’s test for multiple comparisons was performed to compare frozen tissue with different times of archiving. All statistical tests performed were two-sided, and differences were considered significant at *p* values lower than 0.05.

## Supplementary Materials

Supplementary materials can be found at https://www.mdpi.com/1422-0067/21/3/1096/s1. Supplementary S1: Profiling of the microbiota of FFPE samples, S, and paraffin control, Ctrl. Supplementary S2: Relative abundances of the most abundant taxa in the dataset. Table S1: Relative abundance of the five main phyla in the paraffin control and the FFPE samples before and after subtraction of the OTUs from the dataset.

## Abbreviations

FFPE	Formalin-fixed and paraffin-embedded
NGS	Next-generation sequencing
OTU	Operational taxonomic units

## References

[1] Marchesi J.R., Ravel J. (2015). The vocabulary of microbiome research: A proposal. Microbiome.

[2] Huttenhower C., Gevers D., Knight R., Abubucker S., Badger J.H., Chinwalla A.T., Creasy H.H., Earl A.M., FitzGerald M.G., Fulton R.S. (2012). Structure, function and diversity of the healthy human microbiome. Nature.

[3] Lloyd-Price J., Arze C., Ananthakrishnan A.N., Schirmer M., Avila-Pacheco J., Poon T.W., Andrews E., Ajami N.J., Bonham K.S., Brislawn C.J. (2019). Multi-omics of the gut microbial ecosystem in inflammatory bowel diseases. Nature.

[4] Maruvada P., Leone V., Kaplan L.M., Chang E.B. (2017). The Human Microbiome and Obesity: Moving beyond Associations. Cell Host Microbe.

[5] Ferreira R.M., Pereira-Marques J., Pinto-Ribeiro I., Costa J.L., Carneiro F., Machado J.C., Figueiredo C. (2018). Gastric microbial community profiling reveals a dysbiotic cancer-associated microbiota. Gut.

[6] Ahn J., Sinha R., Pei Z., Dominianni C., Wu J., Shi J., Goedert J.J., Hayes R.B., Yang L. (2013). Human Gut Microbiome and Risk for Colorectal Cancer. JNCI J. Natl. Cancer Inst..

[7] Aviles-Jimenez F., Vazquez-Jimenez F., Medrano-Guzman R., Mantilla A., Torres J. (2014). Stomach microbiota composition varies between patients with non-atrophic gastritis and patients with intestinal type of gastric cancer. Sci. Rep..

[8] Dicksved J., Lindberg M., Rosenquist M., Enroth H., Jansson J.K., Engstrand L. (2009). Molecular characterization of the stomach microbiota in patients with gastric cancer and in controls. J. Med. Microbiol..

[9] Shabihkhani M., Lucey G.M., Wei B., Mareninov S., Lou J.J., Vinters H.V., Singer E.J., Cloughesy T.F., Yong W.H. (2014). The procurement, storage, and quality assurance of frozen blood and tissue biospecimens in pathology, biorepository, and biobank settings. Clin. Biochem..

[10] Blow N. (2007). Tissue issues. Nature.

[11] Carrick D.M., Mehaffey M.G., Sachs M.C., Altekruse S., Camalier C., Chuaqui R., Cozen W., Das B., Hernandez B.Y., Lih C.J. (2015). Robustness of Next Generation Sequencing on Older Formalin-Fixed Paraffin-Embedded Tissue. PLoS ONE.

[12] Hedegaard J., Thorsen K., Lund M.K., Hein A.M., Hamilton-Dutoit S.J., Vang S., Nordentoft I., Birkenkamp-Demtroder K., Kruhoffer M., Hager H. (2014). Next-generation sequencing of RNA and DNA isolated from paired fresh-frozen and formalin-fixed paraffin-embedded samples of human cancer and normal tissue. PLoS ONE.

[13] Robbe P., Popitsch N., Knight S.J.L., Antoniou P., Becq J., He M., Kanapin A., Samsonova A., Vavoulis D.V., Ross M.T. (2018). Clinical whole-genome sequencing from routine formalin-fixed, paraffin-embedded specimens: Pilot study for the 100,000 Genomes Project. Genet. Med..

[14] Allaband C., McDonald D., Vazquez-Baeza Y., Minich J.J., Tripathi A., Brenner D.A., Loomba R., Smarr L., Sandborn W.J., Schnabl B. (2019). Microbiome 101: Studying, Analyzing, and Interpreting Gut Microbiome Data for Clinicians. Clin. Gastroenterol. Hepatol..

[15] Knight R., Vrbanac A., Taylor B.C., Aksenov A., Callewaert C., Debelius J., Gonzalez A., Kosciolek T., McCall L.I., McDonald D. (2018). Best practices for analysing microbiomes. Nat. Rev. Microbiol..

[16] Apopa P.L., Alley L., Penney R.B., Arnaoutakis K., Steliga M.A., Jeffus S., Bircan E., Gopalan B., Jin J., Patumcharoenpol P. (2018). PARP1 Is Up-Regulated in Non-small Cell Lung Cancer Tissues in the Presence of the Cyanobacterial Toxin Microcystin. Front. Microbiol..

[17] Bundgaard-Nielsen C., Baandrup U.T., Nielsen L.P., Sorensen S. (2019). The presence of bacteria varies between colorectal adenocarcinomas, precursor lesions and non-malignant tissue. BMC Cancer.

[18] Emery D.C., Shoemark D.K., Batstone T.E., Waterfall C.M., Coghill J.A., Cerajewska T.L., Davies M., West N.X., Allen S.J. (2017). 16S rRNA Next Generation Sequencing Analysis Shows Bacteria in Alzheimer’s Post-Mortem Brain. Front. Aging Neurosci..

[19] Stewart C.J., Fatemizadeh R., Parsons P., Lamb C.A., Shady D.A., Petrosino J.F., Hair A.B. (2019). Using formalin fixed paraffin embedded tissue to characterize the preterm gut microbiota in necrotising enterocolitis and spontaneous isolated perforation using marginal and diseased tissue. BMC Microbiol..

[20] Imrit K., Goldfischer M., Wang J., Green J., Levine J., Lombardo J., Hong T. (2006). Identification of bacteria in formalin-fixed, paraffin-embedded heart valve tissue via 16S rRNA gene nucleotide sequencing. J. Clin. Microbiol..

[21] Prentice L.M., Miller R.R., Knaggs J., Mazloomian A., Aguirre Hernandez R., Franchini P., Parsa K., Tessier-Cloutier B., Lapuk A., Huntsman D. (2018). Formalin fixation increases deamination mutation signature but should not lead to false positive mutations in clinical practice. PLoS ONE.

[22] Kim S., Park C., Ji Y., Kim D.G., Bae H., van Vrancken M., Kim D.-H., Kim K.-M. (2017). Deamination Effects in Formalin-Fixed, Paraffin-Embedded Tissue Samples in the Era of Precision Medicine. J. Mol. Diagn..

[23] Edgar R.C. (2013). UPARSE: Highly accurate OTU sequences from microbial amplicon reads. Nat. Methods.

[24] Edgar R.C. (2016). SINTAX: A simple non-Bayesian taxonomy classifier for 16S and ITS sequences. bioRxiv.

[25] Lozupone C., Lladser M.E., Knights D., Stombaugh J., Knight R. (2011). UniFrac: An effective distance metric for microbial community comparison. ISME J..

[26] Caporaso J.G., Kuczynski J., Stombaugh J., Bittinger K., Bushman F.D., Costello E.K., Fierer N., Peña A.G., Goodrich J.K., Gordon J.I. (2010). QIIME allows analysis of high-throughput community sequencing data. Nat. Methods.

