# Supplementary material for: Evaluation of the Use of Formalin-Fixed and Paraffin-Embedded Archive Gastric Tissues for Microbiota Characterization Using Next-Generation Sequencing
Source: Int J Mol Sci. 2020 Feb 7;21(3):1096. doi: 10.3390/ijms21031096 (PMC7037826; doi:10.3390/ijms21031096)
Supplement: Supplementary file 1 [file ijms-21-01096-s001.pdf]

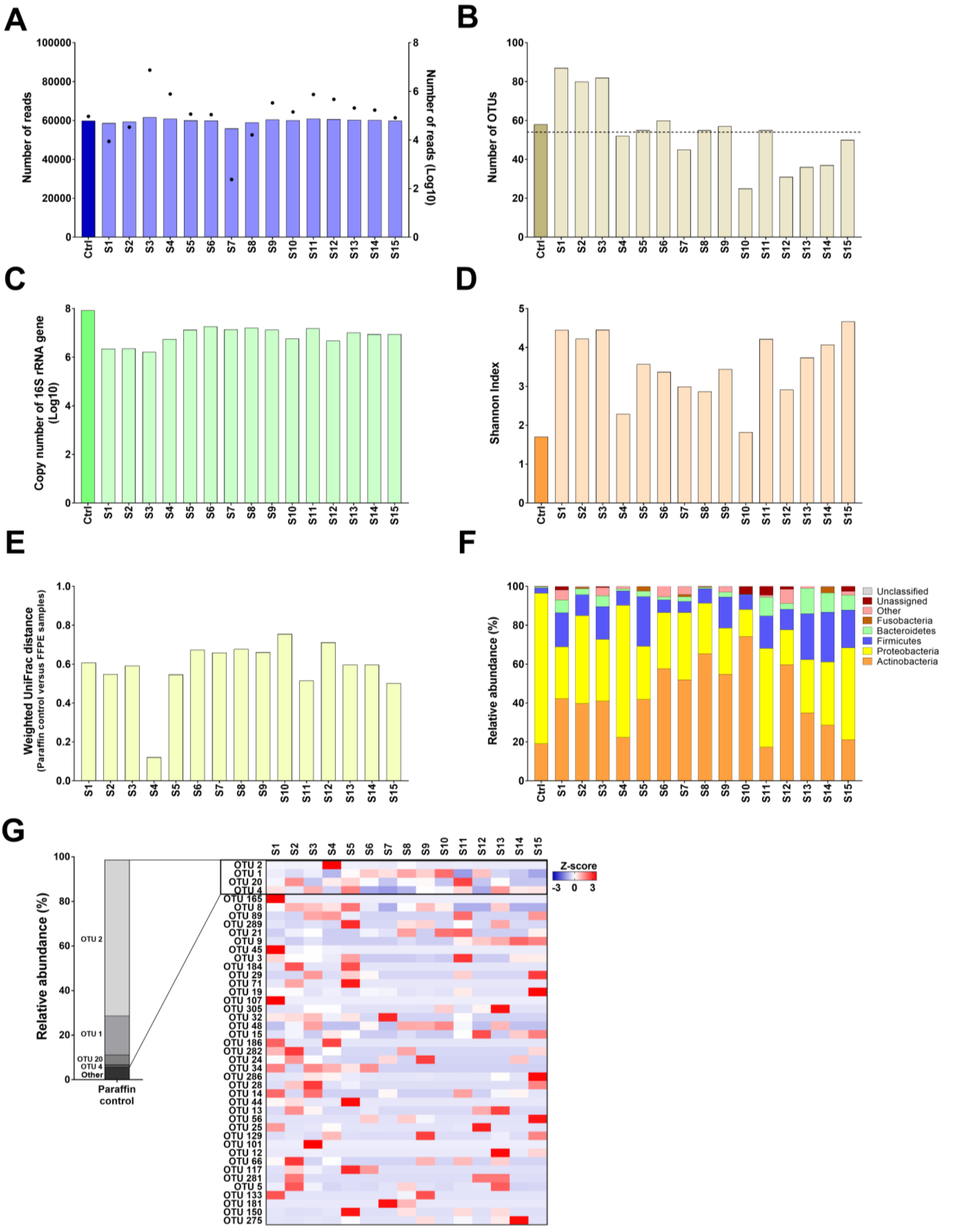

Supplementary Figure 1. Profiling of the microbiota of FFPE tissue samples, S, and of paraffin control, Ctrl**.** (A) Total number of reads after sequencing and quality filtering. The absolute number of reads is represented by black dots, and their log10 value are represented by blue bars. (B) Number of OTUs was obtained after randomly subsampling to a fixed depth. The line stands for the average number of OTUs in the dataset. (C) Total bacterial load determined using universal 16S rRNA qPCR assay by sample. (D) Alpha-diversity estimated by the Shannon index and (E) beta-diversity calculated by weighted UniFrac distance between FFPE samples and paraffin control. (F) Relative abundance of phyla. (G) OTU-based analysis, showing the relative abundance of OTUs of the paraffin control in each of the FFPE tissue samples.

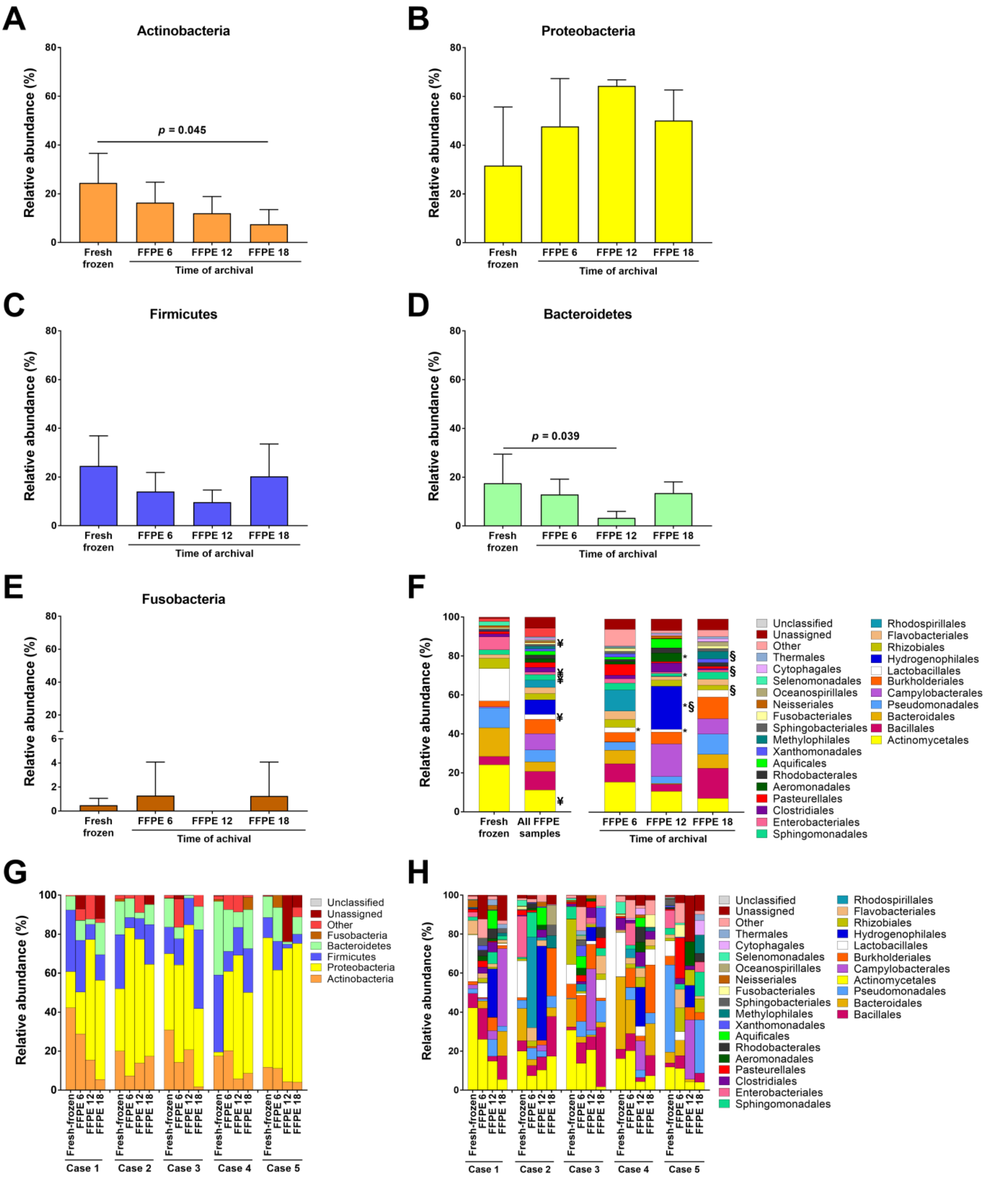

Supplementary Figure 2. Relative abundances of the most abundant taxa in the dataset. (A-E) Relative abundances of main phyla in paired fresh-frozen and FFPE specimens obtained after 6, 12, and 18 months of archival. (F) Relative abundance of orders of paired fresh-frozen tissue, all FFPE tissue samples, and FFPE tissue samples obtained at different times of archival. ¥ and * stand for significantly different from fresh-frozen tissues, and § stands for significantly different between times of archival at $p < 0.05$. (G-H) Relative abundances of (G) phylum and (H) order, per individual case.

Supplementary Table 1. Relative abundance of the five main phyla in the paraffin control and in the FFPE samples before and after subtraction of the OTUs from the dataset.

Phyla	Paraffin control (%)	FFPE samples before OTU subtraction (%)	FFPE samples after OTU subtraction (%)	Fresh-frozen samples (%)
Actinobacteria	19.1	43.6	11.9	24.5
Proteobacteria	77.4	33.4	54.1	31.6
Firmicutes	2.67	14.5	14.7	24.6
Bacteroidetes	0.56	4.70	9.90	17.5
Fusobacteria	0.22	0.50	0.85	0.49
Other	0.06	2.35	4.88	1.12
Unassigned	0.03	0.99	3.71	0.17
Unclassified	0	0.003	0.006	0.02
